# Letter from the World Association for Animal Production

**DOI:** 10.1093/af/vfab011

**Published:** 2021-06-19

**Authors:** Philippe Chemineau

**Affiliations:** President WAAP, Hoover, AL, USA

This issue of *Animal Frontiers* on Animal Domestication is the first to be overseen by the World Association of Animal Production (WAAP). It marks the entry of this federation into the consortium that owns *Animal Frontiers* magazine. I would like to thank here the founding members of the consortium, in particular ASAS and EAAP, for welcoming us, thereby showing a common desire to give this magazine a more global dimension and to contribute to maintaining the high quality of articles published there for more than 10 yr.

This magazine aims to be an interface between the world of science and its users, whether they are decision-makers, teachers, technicians, students, and even researchers in the animal sciences sector or in neighboring sectors.

WAAP, like the other members, will be responsible for one annual issue of the magazine. This is a sign that we are now confronted, as scientists interested and committed in animal sciences, with global problems that will have to be dealt with at the places where they arise through active collaboration not only between countries, but also between continents.

The climate emergency is here, the collapse of biodiversity in a large part of the globe is now a reality, the pressure on natural environments has never been so strong and, at the same time, we must feed in a healthy way and economically accessible, an ever-growing population. Like other human activities, agriculture, and in this context animal agriculture, contributes to a portion of these man-made disorders of the modern era. The latter is currently accused of all the evils in the matter of climate change and loss of biodiversity. However, animal agriculture can also be part of the solution by participating in the resolution of climate, biodiversity, and food crises. How to produce better while reducing its carbon impact? How to develop farming systems that protect biodiversity? What diets based on animal products should be promoted for better health?

We will not be able to answer these questions by remaining isolated in our respective countries. We need to exchange and exchange again, first the ideas and then the results of our research, leading finally to the innovations to enable us to answer the questions presented above. Although the current pandemic is not very favorable, it will also be necessary to encourage networking interactions among men and women who are involved in the animal sciences, so that this community gets to know one another and thereby increases our collective scientific and technical effectiveness.

*Animal Frontiers* is one of the tools that allows this flow of information and WAAP is the global network of Animal Science associations that wants to promote exchanges between its members. Current members of the Animal Frontiers Consortium understand and share this strategy and therefore we wish to thank them for giving us the opportunity to serve as a partner in this Consortium.

I am proud and happy that this first issue, which presents the history of animal domestications in the Neolithic and later, is the crucible of this rapprochement between *Animal Frontiers* and our federation.

## About the Author

**Philippe Chemineau** is initially an Agronomist. He has a PhD in Reproductive physiology of domestic animals. He is Emeritus Director of Research INRAE (France) and President World Association of Animal Production (WAAP). He has been Head of the INRAE Division Animal Physiology and Livestock Systems; Member of national INRAE Management Board; Head of the « Delegation for scientific expertise, Foresight and Advanced studies (DEPE)” INRAE Paris; Head of the INRAE “ Direction of Regional policy, Higher education and Europe (DARESE)”. He has also been President of EAAP (European Association of Animal Production), the European Federation of Animal Science. He has published 196 publications and has an h-index: 38.

